# Connecticut Valley Dental Society

**Published:** 1883-12

**Authors:** 


### CONNECTICUT VALLEY DENTAL SOCIETY.

TWENTIETH ANNUAL MEETING, HELD AT THE HAYNES HOUSE, SPRINGFIELD, MASS., NOVEMBER 7TH AND 8TH, 1883.

The meeting was opened at 2.30 P. M., the President, Dr. Morgan, occupying the chair. After the reception of the regular reports and the transaction of routine business, it was moved that "Incidents of Office Practice" be taken up.

*Dr. Bartholomew*—Said that if life was to be kept in the society the members must contribute more, and not depend entirely upon the eminent men from abroad for papers and instruction. To come here for the sole purpose of listening to strangers was no way to keep up a society. He then related a case of disease of the gums and teeth at the gingival margin, caused he believed by ingredients of a physician's prescription for malaria. He found that sulphuric acid was used as a solvent for the quinine in the prescription. Cessation of the medicine did not stop the softening, but a strong solution of bi-carbonate of soda temporarily checked it.

*Dr. Geo. L. Parmele*—Has been much interested in the reports of some dentists who have visited the hospitals in New York. There is one gentleman who frequently goes to Roosevelt Hospital. He had noticed in the past, that in the treatment of wounds iodoform had been freely used, but at his last visit he had found everything in the amphitheatre changed. The floor beneath the operating tables was so arranged as to float away the debris, and iodoform is no longer employed, but the new German treatment by corrosive sublimate has been substituted. The solution used is one gram to the thousand grams of water. The patient is placed upon the table, and the wound thoroughly washed by a stream of this solution, instead of by the application of the sponge. After an operation the wound is closed by sutures soaked in the oil of rosemary, and mats of powdered peat soaked in a corrosive sublimate solution are used in the dressing.

*Dr. Thayer*—Did not believe that malaria had any effect upon the teeth. Thought that in the case mentioned by Dr. Bartholomew the sulphuric acid used in the prescription had wrought the mischief. He had learned a better way to treat malaria than by tonics. Observation had taught him that the diseased condition was caused by irritation of the spinal nerves, resulting in hepatic congestion. Had used mandrake peptonoids and capsicum, the latter to regulate the drastic effect of the remedy.

*Dr. L. D. Shepard*—Spoke by invitation about his trip abroad during the past summer. He said that no rest was so complete as that of an ocean voyage. It is such an entire change from the home life, and the sea air is so invigorating, and the opportunities for improvement are so great, that in his opinion it is the most sensible way in which one can spend a vacation. He referred to the state of the profession in England, and to the social distinctions there. John Tomes and Spence Bate have lately been honored by the distinction of F. R. S., and this lifts them far above the common level, in the estimation of the world of society, and gives them a social distinction not enjoyed by other dentists.

The election of officers took place at this point, and resulted in the choice of the following:

President—Dr. A. M. Ross, Chicopee, Mass.

1st Vice President—Dr. Geo. L. Parmele, Hartford, Conn.

2nd Vice President—Dr. S. B. Bartholomew, Springfield, Mass.

Secretary—Dr. W. F. Andrews, Springfield, Mass.

Assistant Secretary—Dr. A. Maxfield, Holyoke, Mass.

After the installation of officers elect, the regular order was called.

Section One had no report. Section Two reported through its chairman, that a paper by Dr. J. L. Williams from that section would be read at the evening session, and that Dr. E. A. Stebbins would present a pathological case.

*Dr. E. A. Stebbins*—Presented a case of absorption about the roots of a superior first bicuspid tooth. The patient first came under his care two years before, and the present condition did not develop until some time after he had removed the tartar deposits. When this was first noticed the teeth were loose, the gum tissue turgid, and he could easily reach the apex of the root with an instrument. Sulphuric acid was injected, but since then the tooth had received no treatment. He presented the case here for advice and counsel.

Dr. Searll.—Whether this condition is inherited or is the consequence of neglect, we have no testimony to show. There is no indication of constitutional taint. We have such diseases in their primary and secondary form. The primary is amenable to treatment, but the secondary is inherited, and is comparatively incurable. We are not sufficiently heroic in our treatment of such cases. I scarcely think that this tooth can be permanently benefited by remedies, but there are other teeth in the same mouth that can be saved from the fate of this one. I object to the use of the syringe in the treatment of such cases, because I use strong remedies that should not come in contact with other localities and tissues than the diseased ones. I sometimes make a single application of a solution of one part of sulphuric acid in three of water. At other times I use deliquesced carbolic acid. If it is again necessary to medicate I perhaps employ either of these, but in reduced strength, always applying with a suitably shaped piece of orange wood.

*Dr. Parmele*—Dr. Searll's objections do not apply to the syringe if that of Dr. Farrar is used, and an assistant is called to manipulate the screw-rod.

*Dr. Stebbins*—Wished to ask two questions. Can this condition be defined, and could it be caused by mercury introduced into the system at the age of twelve years?

*Dr. Stockwell*—Detailed cases of this disease, and believed that the cures effected had been due to the antiseptic treatment used.

*Dr. Bartholomew*—Thought this the worst class of cases we have to deal with. They may be divided into two forms: one was wholly local, the other constitutional. The former is readily cured if you can control the patient. He cited the cure of a lady for whom Dr. Riggs operated twelve years ago, and the trouble was thought to be completely overcome. But some time since a disordered condition induced the diseased state again, and now she is losing her teeth, one by one.

*Dr. Shepard*—Said that he had been a faithful student of the symptoms of this disease since he saw Dr. Riggs operate upon Dr. Goodrich, fifteen years ago. If we attribute our failures to constitutional conditions, we are cowards. How far it is best to distrust the presence of constitutional taint and rely upon heroic treatment, is the most important question to be answered. Has seen the father and the child both suffering from this disease. The child was responsive to treatment, the father was not. There was, then, an exciting cause in the parent which did not exist in the child, and that placed him beyond cure. Perhaps it was that in his case the condition had become chronic, and if it had been taken in time it might have been less obstinate. The only treatment that he could recommend in the case in hand would be that of rest, secured by the application of some fixture to act as a splint, and to be allowed to remain as long as the diseased condition continued. A bar of gold or platinum might pass from an anchorage in the cuspid, between the cusps of the diseased tooth, to the second bicuspid. Such a bar would press the tooth to place, and hold it at rest. There was evidently enough of the alveolar process to support the tooth, when the inflammation was subdued.

*Dr. Searll*—Described such an appliance as he had used. It was supported by four clasps, and was removable by the dentist only.

Upon motion the society adjourned until evening.

(To be continued.)

